# Insights into Non-Alcoholic Fatty Liver Disease and Non-Alcoholic Steatohepatitis: The Role of Fibrinogen and Pepsinogen

**DOI:** 10.34172/mejdd.2025.438

**Published:** 2025-10-31

**Authors:** Zahra Eslami, Alireza Norouzi

**Affiliations:** ^1^Department of Clinical Biochemistry, Golestan University of Medical Sciences, Golestan, Iran; ^2^Department of Clinical Biochemistry, Hamadan University of Medical Sciences, Hamadan, Iran; ^3^Golestan Research Center of Gastroenterology and Hepatology, Golestan University of Medical Sciences, Gorgan, Iran; ^4^University of Queensland, Faculty of Medicine, Great Brisbane Medical School, Brisbane, Australia; ^5^Princess Alexandra Hospital, Department of Gastroenterology and Hepatology, Brisbane, Queensland, Australia

**Keywords:** Non-alcoholic steatohepatitis, Non-alcoholic fatty liver disease, Fibrinogen, Pepsinogen, Inflammation, Liver fibrosis

## Abstract

**Background:**

Fibrinogen is a multifunctional protein that contributes to inflammatory processes. Elevated levels of fibrinogen have been associated with increased liver fibrosis and disease severity of non-alcoholic steatohepatitis (NASH). Fibrinogen appears to interact with other factors implicated in NASH pathophysiology, such as activation of immune cells to induce pro-inflammatory cytokines and cellular signaling pathways. Additionally, fibrinogen-mediated pathways may disrupt normal tissue repair processes. Pepsinogen, the inactive precursor of pepsin and a specific functional enzyme in the gastric mucosa, is a serological marker with subtypes closely related to different gastrointestinal diseases. Changes in pepsinogen levels in liver disease are not entirely understood and likely relate to many factors, including inflammation, altered gastrin levels, and *Helicobacter pylori* infection. The clinical implications of altered pepsinogen levels in liver disease are still under investigation, but they may have relevance in the diagnosis and monitoring of liver disease.

**Methods:**

Databases searched included PubMed, Google Scholar, and Scopus from 2005 to 2024, and the following keywords were used: fibrinogen, pepsinogen, non-alcoholic steatohepatitis, NASH, non-alcoholic fatty liver disease, NAFLD, and fibrosis.

**Results:**

As a potential biomarker for NASH, measuring fibrinogen levels could offer valuable insights into disease diagnosis and progression monitoring. On the other hand, pepsinogen is involved in gut health and liver function, contributing to liver inflammation and fibrosis through mechanisms like the gut-liver axis and signaling pathways.

**Conclusion:**

Overall, both fibrinogen and pepsinogen appear to have a more significant negative impact on liver health and the progression of NAFLD than beneficial effects. Integrating assessments of fibrinogen and pepsinogen into clinical practice could facilitate earlier intervention strategies aimed at slowing disease progression.

## Introduction

 Nonalcoholic steatohepatitis (NASH) represents a significant clinical challenge as a progressive form of fatty liver disease characterized by inflammation and liver damage.^1^ In the pathogenesis of NASH, lipid accumulation, inflammation, and oxidative stress play critical roles in driving disease progression. They have drawn significant attention in the medical field due to their association with obesity, insulin resistance, and metabolic syndrome.^2^ The prevalence of nonalcoholic fatty liver disease (NAFLD) has been rising globally, affecting approximately 30% of the general population^3^, while NASH affects around 3%-5% of individuals worldwide.^4^ Understanding the link between fibrinogen levels and NAFLD/NASH is crucial in shedding light on potential biomarkers for disease progression and severity. Recent research has highlighted the potential involvement of fibrinogen, a key protein in blood clotting and inflammation, in the development and management of NAFLD/NASH.^5^ Fibrinogen is a key plasma glycoprotein synthesized by hepatocytes involved in blood clotting through its conversion into fibrin during coagulation.^6^ However, beyond its traditional role in hemostasis, emerging evidence suggests that fibrinogen plays a role in inflammatory processes and contributes to the pathogenesis of various diseases including cardiovascular disease (CVD) and liver disorders.^7-9^ Previous studies have indicated an association between fibrinogen levels and chronic liver diseases such as hepatitis C infection and cirrhosis.^10-12^

 Additionally, pepsinogen, an inactive precursor of pepsin primarily secreted by gastric chief cells,^13^ plays a crucial role in the pathogenesis of NAFLD and its progressive form, NASH. While there is limited direct evidence linking pepsinogen to NAFLD/NASH, previous studies have highlighted the intricate interplay between gut health, intestinal permeability, and liver function. The gut-liver axis is a key pathway through which factors like bile acids, gut microbiota metabolites, and inflammatory mediators can influence hepatic lipid metabolism and inflammation. Disruption of this axis can lead to hepatic steatosis and inflammation characteristic of NAFLD/NASH.^14-16^ Understanding how pepsinogen levels or activity may impact gut health and subsequently contribute to NAFLD/NASH development warrants further investigation for a comprehensive understanding of the disease pathogenesis.^17^

## Fibrinogen Plays a Role in the Pathogenesis of NAFLD/NASH

 Fibrinogen interacts with various molecular components within hepatic cells to modulate key cellular processes involved in NAFLD pathogenesis. It can activate pro-inflammatory pathways leading to hepatic inflammation and fibrosis, ultimately exacerbating liver damage in individuals with NAFLD.^6^ Moreover, fibrinogen may exacerbate inflammation within the liver through interactions with immune cells such as macrophages and neutrophils.^7^ By activating inflammatory pathways or modulating cytokine production, fibrinogen can perpetuate hepatic inflammation in NASH patients, contributing to disease severity and complications.^8^ Furthermore, targeting fibrinogen-related pathways pharmacologically could offer novel therapeutic interventions for managing NAFLD progression and reducing associated complications like cirrhosis or hepatocellular carcinoma (HCC).^9^ Otherwise, NAFLD is believed to be a risk factor for CVD because the liver produces increased levels of fibrinogen and promotes thromboembolism through a prothrombotic state. The in vitro model of NAFLD indicated that each of the three constituent subunits of fibrinogen were upregulated, providing evidence that NAFLD alone is a determinant of CVD risk.^10^ Individuals with NAFLD demonstrate increased levels of inflammatory and pro-thrombotic markers, including C-reactive protein, fibrinogen, and plasminogen activator inhibitor-1 (PAI-1), that improve with weight loss. The relationship between NAFLD and thromboembolism is related to primitive thrombotic state and the hypercoagulability state exhibited by NAFLD individuals, related to increased thrombotic factors and a low PAI-1 state.^11^ NAFLD is involved in the pathophysiology of systemic pro-inflammatory/pro-thrombotic state, particularly NAFLD in its inflammatory variety, (NASH) which potentially releases factors produced from the steatotic liver.^12^ These findings highlight the importance of understanding NAFLD-mediated pathways in CVD development and suggest the need for targeted treatment strategies and improved risk prediction methods.

 By disrupting specific interactions between fibrinogen and cellular receptors involved in fibrogenesis or inflammation, researchers may develop targeted therapies that mitigate liver damage and improve patient outcomes. Table 1 was shown studies on fibrinogen in NAFLD/NASH.

**Table 1:** Studies on fibrinogen in NAFLD/NASH

**Authors**	**Summery**	**Main findings**
Assy et al^13^	In a cross-sectional study of 44 NAFLD patients and 10 controls, no significant differences in fibrinogen levels were found between groups, though specific effect sizes were not reported.	1- No significant differences in fibrinogen levels were reported among patients with fatty liver, non-alcoholic steatohepatitis, chronic hepatitis, or the healthy control group 2- Neither mean fibrinogen levels nor effect sizes nor correlation coefficients with hepatic fibrosis 3- Other thrombotic markers, such as protein S and protein C, were associated with fibrosis
Yeung et al^10^	This study investigates the individual contribution of NAFLD to CVD risk factors using an in-vitro model, finding that NAFLD is an isolated determinant of CVD due to the up-regulation of fibrinogen, a pro-coagulant molecule.	1- The study found that fibrinogen alpha, beta, and gamma chains were significantly upregulated in cells subjected to nutrient overload, a model for NAFLD. 2- This up-regulation provides a possible mechanism for the excess CVD mortality observed in NAFLD patients by enhancing clot strength. 3- NAFLD is suggested to be an independent risk factor for CVD due to its role in increasing fibrinogen levels, which are associated with a higher risk of thrombosis and CVD.
Colak et al^14^	This study measured plasma fibrinogen-like protein 2 (fgl2) levels in patients with biopsy-proven NAFLD and found that fgl2 levels were significantly higher in patients with definite and borderline NASH compared to controls, suggesting elevated fgl2 levels in more severe forms of NAFLD.	1-Plasma fgl2 levels were significantly higher in patients with definite and borderline NASH compared to controls. 2- No significant differences in fgl2 levels were found between patients with simple steatosis and controls. 3- There were no associations between fgl2 levels and the fibrosis stage or steatosis grade.
Taylor et al^15^	A meta-analysis of 13 studies involving 4428 NAFLD patients found that increasing fibrosis stage is associated with higher risks of mortality and liver-related morbidity, with consistent findings even after adjusting for confounders, but inconsistent effects on quality of life.	1-The study found that increasing stages of fibrosis are associated with higher risks of all-cause mortality, liver-related mortality, liver transplant, and liver-related events in NAFLD patients. 2- The risk ratios remained significant after adjusting for confounders such as age and sex, particularly in patients with NASH. 3- Biopsy-confirmed fibrosis is associated with increased risks of mortality and liver-related morbidity in NAFLD patients, both with and without NASH.
Xin et al^16^	The study established a peptidomics pattern to distinguish NAFLD patients from their twin controls, identifying Fibrinopeptide A and Complement C3f as potential diagnostic markers or therapeutic targets for NAFLD.	1-Eleven peptides were upregulated and seven peptides were down-regulated in the NAFLD group compared to healthy controls. 2-Complement C3f and fibrinopeptide A had the highest ROC values for distinguishing NAFLD cases from controls and were significantly higher in the NAFLD group. 3- The study established a peptidomics pattern that can help distinguish NAFLD patients from their twin controls, with potential diagnostic markers being complement C3f and fibrinopeptide A.

## Intracellular Signaling Cascades of Binding Fibrinogen to Integrins

 Integrins are transmembrane receptors that are heterodimers and mediate cell-adhesion. Most integrins, with their head region of the extracellular domains bind to extracellular matrix (ECM) glycoproteins like laminins and collagens found in basement membranes or connective tissue components like fibronectin.^17^ The exact mechanisms underlying the involvement of fibrinogen in NAFLD pathogenesis are multifaceted. One proposed mechanism involves fibrinogen binding to integrins on hepatic stellate cells (HSCs). Fibrinogen binding to integrins on HSCs initiates crucial intracellular signaling cascades that contribute to liver fibrogenesis.^18^ Integrins, through interactions with specific collagen molecules in the ECM, induce conformational changes in latent transforming growth factor-beta (TGF-β), facilitating its activation and promoting HSC activation. Additionally, ECM1 has been identified as a stabilizer of latent TGF-β within the liver ECM by interacting with αv integrins, leading to downstream signaling events that trigger HSC activation and ultimately liver fibrosis progression.^19^ These findings underscore the pivotal role of fibrinogen-integrin interactions in modulating signaling events associated with hepatic fibrosis, emphasizing their relevance as potential therapeutic targets for combating NASH-associated hepatic fibrosis.^20^ Additionally, fibrinogen may interact with other cellular receptors or growth factors present in the hepatic microenvironment to further exacerbate fibrotic responses.^21^ However, it is believed that systemic inflammation triggered by excess adiposity may drive an increase in hepatic synthesis of acute-phase proteins like fibrinogen.^22^ Moreover, insulin resistance commonly observed in NAFLD/NASH can stimulate hepatic production of fibrinogen via inflammatory mediators such as interleukin-6 (IL-6).^2,23^ Furthermore, recent studies suggest that fibrinogen may serve as an independent predictor of NAFLD severity. Elevated levels of this protein have been associated with advanced fibrosis stages and increased risk for progression to cirrhosis among patients with NASH.^24,25^

## HFREP1 Signaling Cascades in Liver

 HFREP1, a novel hepatokine, also known as HUWE1, MULE or I-Rec pathway and plays a significant role in the pathogenesis of NAFLD and type 2 diabetes.^26^ The activation of the I-Rec pathway regulates hepatic lipid metabolism through sterol-regulatory element-binding protein-1c (SREBP1c), which is crucial for upregulating genes involved in fatty acid biosynthesis. When NAFLD-related hepatic insulin resistance occurs, inhibition of the I-Rec molecular pathway leads to a lack of hepatic gluconeogenesis inhibition while leaving de novo lipogenesis unaltered or even increased. This dysregulation further exacerbates NAFLD progression towards more severe stages like NASH through Akt/FoxO1 inhibition and sustaining SREBP1c activity.^27^

 Studies have shown that HFREP1 regulates hepatocyte growth and proliferation through various signaling pathways, including the activation of ERK1/2 cascade. The activation of ERK1/2 by HFREP1 not only induces hepatocyte proliferation but also influences cell metabolism. While ERK1/2 is activated by insulin, its increased activity can contribute to the development of insulin resistance. HFREP1 responds to inflammatory cytokines like interleukin-6, highlighting its involvement in inflammation-induced responses.^28^ This complex interplay between HFREP1 and intracellular signaling cascades underscores its significance as a key player in the pathogenesis of insulin resistance and type 2 diabetes.

 HFREP1 plays a crucial role in liver function by regulating the degradation of proteins like transferrin receptor (TfR1) and suppressing ferroptosis in acute liver injury. The interaction between HUWE1 and TfR1 in hepatocytes, highlighting the significance of the HUWE1-TfR1 axis in hepatic ischemia/reperfusion injury. Additionally, it had been shown that loss of Huwe1 in hepatocytes can exacerbate acute liver injury by leading to elevated expression of pro-inflammatory cytokines like TNF-α and IL-6.^29^ Interestingly, research on mice with specific deletion of Huwe1 in cardiomyocytes has unveiled broader implications beyond liver function, revealing its involvement in maintaining cellular antioxidant systems and preventing cardiac hypertrophy and dysfunction.^30^

## Intracellular Signaling Cascades Involved in the Activation of Pepsinogen in Liver Cells

 In liver cells, the activation of pepsinogen involves intricate intracellular signaling cascades. One key pathway implicated in this process is the TLR signaling pathway. Additionally, HSC activation can be triggered by various cytokines released from different cell types within the liver, such as hepatocytes and macrophages, contributing to the pathological processes observed in conditions like NAFLD.^31,32^ These interconnected signaling pathways highlight the complex interplay between immune responses and hepatic cellular activation mechanisms essential for understanding pepsinogen activation within liver cells. While research on pepsinogen’s direct impact on NAFLD is limited, understanding its role could provide valuable insights into the disease’s pathogenesis. Furthermore, investigating the association between pepsinogen levels and insulin resistance in NAFLD patients may shed light on additional mechanisms underlying the development of this increasingly prevalent liver disorder.

## Contribution of Pepsinogen in the Development and Progression of NAFLD

 Pepsinogen has been implicated in the development of NAFLD.^33^ Research suggests that immune responses within the liver microenvironment play a crucial role in this process.^34^ Pepsinogen may contribute to liver inflammation and fibrosis, pivotal processes in advancing NAFLD to more severe stages like NASH and liver cirrhosis.^33^

 Toll-like receptors (TLRs), particularly TLR4, have been identified as key factors activating HSC involved in liver fibrosis progression.^35^ Moreover, upregulation of TLR2 during inflammatory conditions highlights the involvement of TLRs in mediating cellular responses within the liver, further emphasizing their impact on NAFLD pathogenesis.^32^ The interconnected signaling pathways between immune responses and hepatic cellular activation mechanisms underscore the complex nature of pepsinogen activation and its implications in NAFLD pathophysiology.^31^

 The connections between pepsinogen, microbiota, and gastric health have been described in a number of studies published to date. A lower microbial richness in the upper digestive tract was associated with a lower ratio of pepsinogen I/II and presence of esophageal squamous dysplasia in a study that suggests a possible contribution of the microbiota to the development of gastric and esophageal cancer.^36^ Elevated pepsinogen A3 levels in the trachea were associated with higher bacterial and fungal richness, changed microbial composition and higher levels of inflammatory markers.^37^ Some serological markers, such as pepsinogen levels and/o r *H. pylori* antibodies predicted the number of abundance of potential gastric cancer-associated bacteria.^38^ Notably, *H. pylori* infection and low pepsinogen I combined to pose a risk of distal gastric adenocarcinomas.^39^ These studies exemplify the complex relationship between pepsinogen, microbiota and gastric health, provide a basis for future investigations into these relationships and the role of microbiota in cancer development.

 Recent study has primarily associated serum pepsinogen II (PGII) levels with gastric cancer, where lower levels are indicative of the disease. However, emerging research has begun to explore the potential link between PGII and NAFLD. Notably, individuals with gastric cancer displayed significantly reduced plasma levels of the pepsinogen I/II ratio compared to controls.^40^ Investigating the correlation between serum PGII levels and NAFLD could offer valuable insights for early detection and monitoring of both conditions simultaneously. Understanding this relationship may aid clinicians in identifying individuals at risk for both diseases, facilitating timely intervention and management strategies.

 Fibrinogen and pepsinogen have rarely been investigated for their association with NAFLD progression. The only study to date to measure plasma fibrinogen ‐ like protein 2 (fgl2), used ELISA to measure fgl2. The authors reported fgl2 values at 788 ± 190 pg/mL in definite NASH, 710 ± 140 pg/mL in borderline NASH, 649 ± 162 pg/mL in simple steatosis, and 515 ± 174 pg/mL in controls. They found that fgl2 concentrations were statistically significantly greater in both definite and borderline NASH than in controls (p < 0.001), but did not find a clear association to fibrosis stage or steatosis grade.^14^ Fibrinogen contributes to tissue fibrosis through a variety of interdependent mechanisms. Using models of muscle, kidney, and central nervous system fibrosis, fibrinogen targets cell surface receptors (e.g., CD11b/CD18, αvβ3 integrin, toll-like receptors, intercellular adhesion molecule 1) to stimulate signaling that activates fibroblasts and promotes collagen deposition. Several studies report that fibrinogen engages the TGF-β pathways (e.g., SMAD2 phosphorylation) either by directly stimulating TGF-β production or by making latent TGF-β available as a carrier to promote extracellular matrix deposition. Inflammatory signaling, on the other hand, is also evident as fibrinogen activates host receptors (e.g., CD18 and TLR4) to produce soluble cytokines/chemokines, in one study, 100 times more than monocyte chemoattractant protein 1.^41^ Furthermore, incorporation of fibrinogen into the extracellular matrix is likely modified by fibronectin-dependent assembly and via tissue transglutaminase crosslinking.^42^ Thus, the studies collectively provide evidence for fibrinogen’s multi-faceted role in promoting fibrosis both through direct receptor-mediated effects, TGF-β–dependent fibroblast activation, inflammatory mediator production, and matrix stabilization.^41^

 Diagnosing NAFLD currently relies on imaging modalities like ultrasound and magnetic resonance imaging (MRI), as well as serum biomarkers such as alanine transaminase (ALT) levels and calculation of non-invasive scoring systems like FIB-4 index or NFS score. However, these diagnostic methods have limitations regarding sensitivity and specificity for detecting early stages of NAFLD or differentiating between disease subtypes like NASH.^43^ Understanding how fibrinogen signaling pathways contribute to liver fibrosis could offer new biomarkers or imaging targets that enhance diagnostic accuracy and prognostication for patients with NAFLD.

 In terms of treatment strategies for NAFLD targeting fibrinogen signaling pathways, several approaches show promise based on preclinical studies and emerging clinical trials. Inhibiting specific receptors involved in mediating fibrinogen effects on HSC stellate cells could attenuate their pro-fibrotic behavior and slow down disease progression.^7,44^ Modulating downstream signaling molecules activated by fibrinogen binding may also offer therapeutic benefits by disrupting key events driving liver fibrosis cascade.^45,46^

## Discussion

 Research has shown that fibrinogen levels are associated with the risk factors for metabolic syndrome and NAFLD, independent of overweight and acute inflammation markers like ferritin. In NAFLD patients, there is a dysregulation in iron metabolism characterized by increased hepcidin levels leading to decreased ferroportin expression, resulting in iron retention and subsequent oxidative stress and inflammation.^47^ Additionally, systemic inflammation marked by high-sensitivity C-reactive protein (hs-CRP) has been linked to histological lesions of steatosis and NASH, indicating its predictive value for disease progression.^48^ Therefore, fibrinogen may contribute to liver inflammation in NAFLD by promoting inflammatory responses mediated through interactions with iron metabolism dysregulation and systemic inflammation markers like hs-CRP. On the other hand, pepsinogen has also emerged as a potential contributor to liver inflammation and fibrosis in NAFLD.^49^ A study on gastric biomarkers in high-risk populations for gastric cancer found a correlation between pepsinogen C expression and phenotypic markers of gastric cancer.^50^ Additionally, assessing serum pepsinogen alongside anti-*Helicobacter pylori* IgG was found to be valuable in low-prevalence countries for diagnosing *H. pylori* infection. This suggests that serum pepsinogen levels may serve as a potential indicator of liver health and disease severity in individuals with NAFLD.^51^

 Serum pepsinogen levels have been studied in comparison to fibrinogen levels for accurately identifying NAFLD in individuals. Research by G. Tarantino et al. indicated that inflammatory markers like fibrinogen were analyzed alongside serum concentrations of interleukin-15 (IL-15) and other markers in obese patients with NAFLD. The study found that IL-15 levels, along with age, were independent predictors of early atherosclerosis in this population, showcasing the potential role of cytokines like IL-15 in the atherosclerosis process.^52^ This highlights the significance of exploring various serum biomarkers, including pepsinogen levels, to enhance the diagnostic accuracy and understanding of NAFLD among individuals.

 Similarly, Fibrinogen-like proteins affect metabolic dysfunction-associated steatotic liver disease (MASLD) development through various pathways. One study shows fgl2 induces NF-kB, p38-MAPK and NLRP3-inflammasome signaling via toll-like receptor 4 interactions on macrophages. This inflammatory signaling activates processes resulting in increased pro-inflammatory cytokines, increased reactive oxygen species, and altered lipid metabolism, further increasing steatosis and inflammation, respectively.^53^ Another study noted fgl1 is inhibited in MASLD, however, fgl1 knockout mice have worse liver injury and glucose tolerance but no change to steatosis or fibrosis.^54^ Another review shows that liver sinusoidal endothelial cell dysfunction, platelet activation, and neutrophil extracellular traps (NETs) initiate vascular and immune-mediated injury in MASLD. No studies reported mechanistic data connecting pepsinogen to MASLD development.^55^ Overall, studies indicate fibrinogen-related proteins impact inflammatory signaling while regulating metabolism in MASLD and that pepsinogen has no literature connected.^53^

## Practical Challenges

 Increased levels of fibrinogen are not unique to NAFLD/NASH among inflammatory illnesses, metabolic disorders, and even upon normal physiologic conditions,^56^ therefore, it is not the most sensitive marker of identifying patients with advanced fibrosis or NASH.^57^ Some studies have looked into pepsinogen levels in NAFLD/NASH, but it is not often said to be as a specific or sensitive marker. As pepsinogen has limited specificity and sensitivity in NAFLD/NASH, this makes it less useful for clinical diagnosis or prognosis.^58^

## Conclusion

 Elevated plasma fibrinogen levels have been correlated with the severity of liver fibrosis in patients with NAFLD/NASH. Pepsinogen levels may serve as biomarkers for inflammatory states. It potentially affects gut-liver axis homeostasis or reflecting broader systemic inflammatory states. Indeed, fibrinogen plays a more direct role in the progression of NAFLD and NASH by promoting inflammation and fibrogenesis, whereas the role of pepsinogen is still not well-defined and likely more indirect. There is less direct evidence linking pepsinogen to NAFLD/NASH progression compared with fibrinogen, but changes in digestive enzyme profiles could reflect systemic metabolic disturbances associated with these liver diseases. Although fibrinogen and pepsinogen may provide benefit to a more global assessment of NAFLD/NASH, they are not very specific or sensitive markers for these diseases. Their limitations include largely non-specificity, confounding by additional factors, and the limited utility of clinical diagnosis or prognosis. Further research is needed to elucidate the role of pepsinogen and fibrinogen and their therapeutic potential with high sensitivity and specificity in NAFLD/NASH.
